# Supplementary material for: Identification of photoperiod-induced specific miRNAs in the adrenal glands of Sunite sheep (Ovis aries)
Source: Front Vet Sci. 2022 Jul 22;9:888207. doi: 10.3389/fvets.2022.888207 (PMC9354845; doi:10.3389/fvets.2022.888207)
Supplement: Supplementary file 1 [file Data_Sheet_1.ZIP › Supplementary file/miRDeep2 file.zip!/miRDeep2 file/Know_Structure/LP42A1/oar-mir-432.pdf]

miRBase precursor	: oar-mir-432
Total read count	: 1115
oar-miR-432 read count	: 1108
remaining reads	: 7

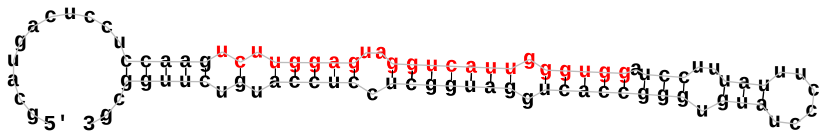

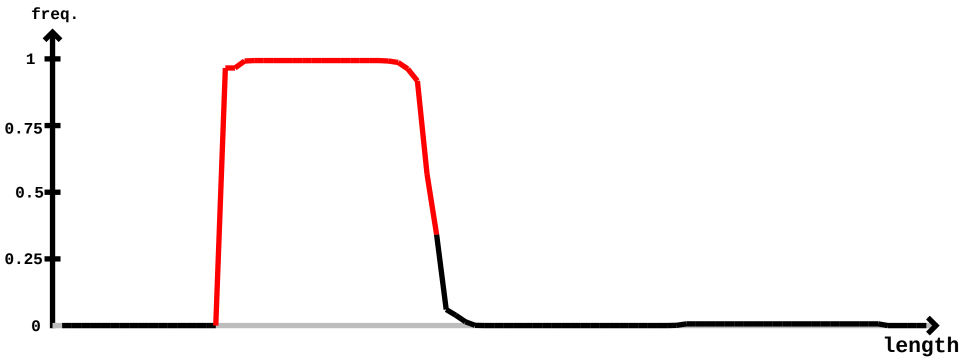


```
                oar-miR-432
5'- gcaugacuccuccaagucuuggaguaggucauugggugggauccuuuauuucccuaugugggccacuggauggcuccuccaugucuuggcg   -3'    exp
    ...........((((.(.(((((..(((((((.(((((.(((..(((......))).)))))))).))))))).))))).).)))))..   reads     mm       sample
    ................ucuuggaguaggucauA.........................................................   1         1        seq
    ................ucuuggaguaggucauu.........................................................   1         0        seq
    ................ucuuggaguaggucauug........................................................   6         0        seq
    ................ucuuggaguaggucauugg.......................................................   27        0        seq
    ................ucuuggaguaggucauuggg......................................................   50        0        seq
    ................ucuuggaguaggucauugggu.....................................................   389       0        seq
    ................ucuuggaguaggucauugggug....................................................   254       0        seq
    ................ucuuggaguaggucauugggugg...................................................   312       0        seq
    ................ucuuggaguaggucauuggguggа..................................................   22        0        seq
    ................ucuuggaguaggucauuggguggau.................................................   13        0        seq
    ................ucuuggaguaggucauuggguggauc................................................   2         0        seq
    ..................uuggaguaggucauugggugg...................................................   1         0        seq
    ..................uuggaguaggucauuggguggа..................................................   2         0        seq
    ..................uuggaguaggucauuggguggau.................................................   12        0        seq
    ..................uuggaguaggucauuggguggauc................................................   13        0        seq
    ..................uuggaguaggucauuggguggaucc...............................................   1         0        seq
    ...................uggaguaggucauuggguggau.................................................   2         0        seq
    ...............................................................acuggauggcuccuccaugucu.....   1         0        seq
    ................................................................cuggauggcuccuccaugucu.....   6         0        seq
```
